# Addressing the HIV/AIDS investment gap through stronger public financial management systems: a human-centered approach

**DOI:** 10.1186/s12913-024-11324-1

**Published:** 2025-05-28

**Authors:** Carlyn Mann, Elan Reuben, Susanna Baker, Mai Hijazi, A. K. Nandakumar, Padma Shetty, Rob Stanley, Onyeka Igboelina, Godfrey Nyombi, Dhimn Nzoya, Samson Oli, Garoma Kena, Carolina Piña, Jordan Tuchman, Kenneth Sklaw, Abimbola Kola-Jebutu, Ivana Lohar, Anh Nguyen Thi Cam, Simplice Takoubo Kamdem, Yves Maxime Kouadio Kouadio

**Affiliations:** 1 Office of HIV/AIDS, Global Health Bureau, U.S. Agency for International Development, Washington D.C, USA; 2 Office of U.S. Global AIDS Coordinator, Washington D.C, USA; 3 Office of Infectious Disease, Global Health Bureau, U.S. Agency for International Development, Washington D.C, USA; 4 Health Office, USAID Tanzania, U.S. Agency for International Development, Dar Es Salaam, Tanzania; 5 Office of HIV/AIDS and Tuberculosis, USAID Nigeria, U.S. Agency for International Development, Abuja, Nigeria; 6 Health Office, USAID Kenya, U.S. Agency for International Development, Nairobi, Kenya; 7 Health Office, USAID Ethiopia, U.S. Agency for International Development, Addis Ababa, Ethiopia; 8 Health & HIV/AIDS Office, USAID Uganda, U.S. Agency for International Development, Kampala, Uganda; 9 Health Office, USAID Dominican Republic, U.S. Agency for International Development, Santo Domingo, Dominican Republic; 10 Health Office, USAID, U.S. Agency for International Development, Windhoek, Namibia; 11 Health Office, USAID, U.S. Agency for International Development, Gaborone, Botswana; 12 Office of Health and Family Planning, USAID , U.S. Agency for International Development, Nepal Kathmandu,; 13 Health Office, USAID Vietnam, U.S. Agencies for International Development, Hanoi, Vietnam; 14 Health Office, USAID Côte d’Ivoire, U.S. Agency for International Development, Abidjan, Côte d’Ivoire

**Keywords:** Public financial management, HIV/AIDS, Health financing, Health systems, PEPFAR, Sustainable financing

## Abstract

**Background:**

UNAIDS estimated that US$29 billion will be required by 2025 to meet HIV/AIDS service demands, with 53 percent expected to come from domestic sources. The PEPFAR-funded, USAID-implemented Sustainable Financing Initiative for HIV/AIDS (SFI), starting in 2014, supported domestic resources mobilization efforts and activities to strengthen countries' public financial management (PFM) systems, positively contributing to much-needed increase in domestic resources for health and HIV.

**Program approach:**

SFI was implemented in 12 countries, supporting activities to build the capacity of governments to mobilize domestic resources for HIV, improve budget absorption, and maximize resource use and develop and use evidence for advocacy to increase domestic government funds for HIV/AIDS. SFI measured impact by agreed upon indicators and estimated return on investment (ROI).

**Results:**

Eight countries focused on building capacity to improve budgeting and execution of health and HIV/AIDS funds; five experienced increases in budget allocation and spending. Kenya country governments spent an additional US$180 million and US$8.7 million on health and HIV, respectively. This contributed to US$60 mobilized and spent for every SFI dollar invested. Eight countries focused on using evidence to advocate for more domestic resources for health and HIV/AIDS from government budgets, increase budget execution, and identify areas for efficiency. Cambodia saw an increase in government commitments for ARVs from US$1.5 million annually from 2018–2020 to US$5 million by 2023.

**Lessons learned:**

Robust data are needed for evidence-based advocacy to increase domestic government funding for HIV/AIDS and to strengthen PFM systems for more efficient and effective resource use; institutionalizing capacity building efforts allows for locally-led technical assistance; policy-related work is a multi-year endeavor; PFM success can be stymied by political transitions, political will, and donor commitments; COVID-19 brought new challenges and new opportunities; measurable results can lead to greater impact; and results are not necessarily solely project attributions with possible inflation of ROI estimates given there was no counterfactual.

**Conclusion:**

Strengthening PFM systems can increase domestic resources for health and HIV through increased revenue and improved efficiency; closing the investment gap to end the HIV/AIDS epidemic by 2030.

## Background

The President’s Emergency Plan for AIDS Relief (PEPFAR) funding over the last decade has remained relatively flat [1]. In many countries this has led to either flat or a decline in donor funding, especially as countries transition from low-income to lower-middle and upper-middle income status. UNAIDS estimated low- and middle-income countries would require an investment of US$29 billion by 2025 to meet the demands for HIV/AIDS services and posited that 53 percent of the total should come from domestic sources [2]. These ambitious targets should be reevaluated considering the negative effects of COVID-19 on specific countries and the global economy, counterbalanced with efficiency gains made in the HIV/AIDS response. PEPFAR has pushed countries and programs to achieve results with less resources and focused on governments taking on more fiscal responsibility for their HIV response.

Sustaining the gains made in the fight against HIV/AIDS has become increasingly important. UNAIDS 2030 fast track targets aim for countries to achieve 95–95-95 targets along the clinical cascade to end AIDS as an epidemic (95% of people living with HIV know their status, 95% of those that know their status are on treatment, and 95% of those on treatment are virally suppressed) [3]. Part of sustaining progress made means that a country’s HIV program is sustainably financed through increased domestic public and private resources and/or to use available resources more efficiently. Domestic public resources consist of government spending for HIV/AIDS using funds from revenue generation activities and the percent allocation to the HIV/AIDS program from the total health budget allocation. Domestic private resources consist of private financing and privately provided HIV services.

This paper focuses on work done in 12 countries under a PEPFAR-funded, USAID-led Sustainable Financing Initiative (SFI) for HIV/AIDS. SFI was implemented from 2014–2022 and was dedicated to mobilizing domestic resources for the HIV response [4]. This initiative had three key pillars consisting of public financial management (PFM), private sector engagement (PSE), and financial risk protection and cross-cutting themes of commodity security, advocacy and analytics, and efficiency. The first pillar, PFM, is the backbone to financing a country’s health system and HIV/AIDS program. Strengthening PFM, leads to increased fiscal space that in turn has the potential to lead to an increase in the domestic resource envelope for health and HIV/AIDS programming for the public and private sectors, and ensuring the resources are spent effectively and efficiently.

### Objectives

Many efforts were already underway to strengthen the systems of PFM, but such efforts were not delivering results. SFI’s focus was on the people utilizing the systems. Activities were developed based on each countries own unique, identified needs. identify the specific needs of the country and then development a program of work. Activities conducted under each country were categorized into the three areas under this umbrella of work was: 1) building capacity to increase domestic government resource mobilization, improve budget absorption, and maximize resource use for health and HIV/AIDS; 2) improving use of evidence-based advocacy; and 3) demonstrating measurable impact. Addressing efficiencies in resource use has become even more important as countries faced unexpected rapid economic downturn due to the COVID-19 pandemic. The International Monetary Fund (IMF) estimated a contraction of the global economy by -3.3 percent in 2020, which is a slightly more optimistic view than previous estimates [5–7]. Initially, the global economy rebounds as countries open their economies with 6 percent growth for 2021 [5]. Unfortunately, the recovery from the COVID-19 pandemic has slowed due to Russia’s invasion in Ukraine, rising inflation, and an increase in sovereign debt distress among a growing number of developing countries led to economic slowdown with a decline in global growth to 3.5 percent in 2022 and projected to fall even further to three percent in both 2023 and 2024 [8]. Even prior to the pandemic there was a steady increase in the debt to GDP ratios of countries. The pandemic has exacerbated this situation as countries have borrowed aggressively to address pandemic related issues. COVID-19 has presented a new challenge with the double shock of needing to increase health spending for the response while donor countries and host countries saw a rapid decline in tax revenues [9]. The program of work presented in this paper will highlight the key steps taken to increase domestic resource mobilization (DRM), strengthen PFM systems, and improve the use of evidence for decision-making with the focus on measurable time-bound results with targeted investments. Furthermore, it provides key considerations for COVID-19 impacts and lessons learned.

## Program approach

A well-functioning PFM system is needed for the accountability of efficient and effective use of public resources. Without this, finite resources can be wasted. PFM consists of a set of laws, regulations, policies, and processes used by a government to determine the resource envelope for public sectors, criteria for allocating the public funds, spending the funds, and to monitor how resources are spent and associated results [10]. The program approach to strengthening PFM in SFI-supported countries was to build capacity to improve budgeting and execution and produce and use evidence for advocacy to increase domestic government funds for HIV/AIDS.

### Build capacity to increase domestic government resource mobilization, improve budget absorption, and maximize resource use

Much of the PFM work comes down to the people advocating for funds, managing the allocation and execution of those funds, and using data the system produces to monitor the use of the funds and inform future budgeting. If the people managing this system do not have the capacity to do it effectively then bottlenecks arise from budgeting to spending and monitoring. Investment in the systems to manage public sector funds is important as are the people using the system. Over the last few decades there has been an emphasis on improving PFM systems in many low and middle-income countries but the in-country capacity to use the systems remains limited. SFI emphasized the importance of building capacity of the users of PFM systems to improve budgeting and execution of public health and HIV/AIDS funds. See Table 1 for the specific activities undertaken in each country. The program of work took on different forms, depending on country-specific needs. In Kenya, Uganda, and Nepal, capacity building was institutionalized by developing training materials, tools, and reference guides and training government officials or community-based organizations to improve budget advocacy, resource allocation and execution. In Kenya, training was provided alongside the Kenya School of Government, which is now delivering the bulk of this support. In other countries, like Vietnam, Botswana, and Nigeria, the focus was to build capacity on budget planning, allocation, and expenditure analysis of government officials at the national and sub-national levels.

**Table 1:** program approach and results for the pfm pillar in 12 sfi countries

Country	Partners	Activities	Results	Financial ROI
Kenya	Health Policy Plus	*Build capacity to improve budgeting and execution* - Supported the health budgeting processes for county governments to increase the proportion of counties’ overall budget allocated to health, and improve allocation to key needs, such as HIV, with full execution of those budgets. Program-based budgeting (PBB) is required for counties planning approach - To build local capacity on PBB-related training, SFI developed a curriculum, validated it with stakeholders including the Treasury, and implemented trainings alongside the Kenyan School of Government – now delivering the bulk of support - Deep-dive support in six counties to improve allocation and budget execution *Produce and use evidence for advocacy to increase DRM for HIV/AIDS* - Demonstrate importance to reinstate ARV and other HIV commodity line item in the national budget through high level advocacy and technically supported analyses provided to the Ministry of Health - Worked with county officials during the planning and budgeting process to prioritize and increase funding for health and HIV	*Build capacity to improve budgeting and execution* - 590 health sector and budgeting officials trained across 26 counties - Average county government allocation to health increased from 23% in 2015/16 to 27% in 2017/18, compared to 13% prior to devolution (2013) - Budget execution rates improved from 79% in 2016/17 to 88% in 2017/18 for deep dive counties - At the county level, US$3.361 billion and US$19.5 million was mobilized and allocated to health and HIV, respectively, from 2016/17 to 2020/21 *Produce and use evidence for advocacy to increase DRM for HIV/AIDS* - National budget line item for antiretrovirals was restored in 2015/16 and increased from US$20 million to US$34 million (2019/20). A total of US$80 million spent so far on ARVs and test kits nationally over 5 years - Still low budget absorption because of procurement issues (73% in 2015/16, 79% in 2016/17, 42% in 2017/18, 128% in 2018/19, and 46% in 2019/20)	59.6:1 (PFM-specific work at county levels) 60:1 (reinstating ARV and test kits budget line item and increasing execution of budget over time)
Uganda	Leadership in Public Financial Management (LPFM-II), Uganda Health Systems Strengthening Activity (UHSS)	*Build capacity to improve budgeting and execution* - Tool development and reference materials to be used in future and - Technical assistance to national and sub-national officials to implement PBB - Strengthened the capacity of the Revenue Authority tax administration to collect taxes - Supporting the Ministry of Health (MOH) transition to PBB, which links the budget to results and health outcomes, thereby making it easier to advocate for resources based on known needs *Produce and use evidence for advocacy to increase DRM for HIV/AIDS* N/A	*Build capacity to improve budgeting and execution* - Over 300 government officials trained on PBB - Increased budget execution rates at MoH increased from 80% in 2015/16 to 97% in 2017/18, leading to an additional US$17.4 million in health spending. Uganda’s Revenue Authority increased debt collection revenue by 50% from UGX 49 billion in 2017 to UGX 73 billion Uganda in 2018, an increase of around US$7 million	9:1
Botswana	Africa Collaborative for Health Financing Solutions	*Build capacity to improve budgeting and execution* - Developed and rolled out Financial Management training packages for budget holders and managers at the sub-national and national levels - Developed system to harmonize SHA and NASA; training on this and collaboration with UNAIDS to ensure continuity *Produce and use evidence for advocacy to increase DRM for HIV/AIDS* - Advocated for the Government of Botswana (GOB) to adopt pooled procurement using evidence and information generated from global, regional, and local assessments - Developed and costed a National of HIV/AIDS basic service package (HABSP)	*Build capacity to improve budgeting and execution* - Supported three Training of Trainers workshops to roll out financial management training packages developed to address inefficiencies in Financial Management (FM). 46 key national and regional staff trained but not all were the identified financial managers (some were human resource personnel that sometimes perform financial tasks). This was because many FM’s needed to fulfill procurement duties because of COVID-19 and vaccine campaigns being rolled out - Local institution was going to be used but due to time restrictions the team relied on its own staff and local consultant. Continued mentoring of TOT rolling out this process was minimal - Move toward routinizing resource tracking processes, the GOB assigned staff to resource tracking (who received training and mentoring) and created a budget line to financially support the harmonized resource tracking process *Produce and use evidence for advocacy to increase DRM for HIV/AIDS* - Estimation of resources needed to sustain gains made in the national response, as well as to scale up interventions outlined in the HABSP - A rapid mapping of available resources (government budget estimates, partner commitments/pledges, including local private sector) against the annual needs is undertaken to inform potential funding gap, hence additional resources mobilization required to meet the annual need - A costed HABSP presents the opportunity for a comprehensive roadmap to guide prioritization, resource allocation and programing towards sustaining and advancing the HIV/AIDS national response	n/a
Côte d'Ivoire	Health Systems Strengthening Accelerator	*Build capacity to improve budgeting and execution* - Strengthened the capacity of the public sector to provide oversight and leadership of HIV programs at central and decentralized levels - Lastly, in September 2020 Côte d’Ivoire organized 2 training good governance workshops with participants including representatives from the General Directorate of Health, the National HIV Control Program, Regional and Departmental Directors of Health, and other representatives of the regional and departmental health system. Participants were presented a tool to monitor financial expenditure, comprising a system to code the interventions, strategies, and objectives of the National Health Development Plan, and integrating action plans of the various stakeholders at the decentralized level of the health system. The Implementing mechanism was unable to continue these activities initiated with the Ministry of Health and Public Hygiene due to reduced budgets. However, the 2021–2025 national health development plan (PNDS 2021–2025) currently being validated includes the “BOOST” budgeting and expenditure monitoring tool at the central level of the Ministry of Health, Public Hygiene and Universal Health Coverage *Produce and use evidence for advocacy to increase DRM for HIV/AIDS* N/A	*Build capacity to improve budgeting and execution* - The National HIV Control Program and General Directorate of Health commit to advocate the use of a financing tool to track health expenditures that will help illustrate the financial landscape for HIV and monitor implementation of the HIV program at the national level - Decentralized HIV / AIDS committees are revitalized and a template for developing local operational plans takes into consideration related issues or impacts of the COVID-19 pandemic to help build management and monitoring capacity of the HIV program at decentralized levels and ensure the sustainability of committees - Coordination framework created between the PNLS and the HIV focal points in the health districts and regions following a redefinition of the roles and responsibilities of the latter, for the strengthening of leadership, monitoring and evaluation of the HIV program at regional and departmental levels of the country	n/a
Nepal	Health Policy Plus	*Build capacity to improve budgeting and execution* - Built civil society organizations (CSOs) capacity to improve budgeting and budget advocacy through tool development, trainings and coaching *Produce and use evidence for advocacy to increase DRM for HIV/AIDS* N/A	*Build capacity to improve budgeting and execution* - 28 participants from national CSOs worked with NCASC to develop specific strategic actions to improve budgeting and advocacy for CSO funding by the government - Advocacy agenda developed with key areas of focus, responsible stakeholders, and role of CSOs at national, provincial, and local levels - 24 local CSOs participants worked alongside local government officials to receive training in budget advocacy tool developed; identified key advocacy messages and prioritized strategic actions; CSOs formed HIV advocacy network; enhanced commitments from CSOs and provincial government officials to prioritize HIV interventions and increase budget allocation	n/a
Nigeria		*Build capacity to improve budgeting and execution* - SFI worked with government officials in Rivers, Kano, and Lagos states to improve their budget planning *Produce and use evidence for advocacy to increase DRM for HIV/AIDS* - Technical assistance to help develop the HIV Domestic Resource Mobilization Strategy (DRMS)	*Build capacity to improve budgeting and execution* - US$1.7 million increase in spending in Lagos from - 2016 to 2018, and US$1.0 million in Kano and US$1.7 million in Lagos allocated for the HIV response from 2019 to 2020 - No results in Rivers because during program implementation decided to stop work because lack of political will and no results occurring *Produce and use evidence for advocacy to increase DRM for HIV/AIDS* - DRMS finalized and endorsed	35:1
Vietnam		*Build capacity to improve budgeting and execution* - Trained and provided technical assistance to strengthen the Government of Vietnam’s HIV budget and expenditure analysis, with a focus on ARV procurement and management - Technical assistance to develop financial analysis on SHI Liability to cover HIV services to demonstrating the feasibility of SHI as the primary financing mechanism for the HIV response - Comprehensive report analyzing options for central procurement of drug using SHI fund - Technical assistance to evaluate domestic resources for 2016–2020 HIV strategy and estimate resource need and funding gaps for 2022–2030 HIV strategy	*Build capacity to improve budgeting and execution* - Budget execution and social health insurance (SHI) commitments for ARVs increased by US$15.4 million and US$2 million was saved through improved procurement processes - Targeted activities in four focus provinces, SFI helped local governments customize their copayment subsidy models and update their cost estimates for copayment subsidy needs - At the national level, 52 percent of the provinces receiving technical assistance committed to mobilize funds for SHI premiums and ARV copayments - Local governments committed US$2.4 million to subsidize SHI premiums and copayments for PLHIV	19:1
Namibia	Africa Collaborative for Health Financing Solutions	*Build capacity to improve budgeting and execution* - SFI worked with the Resource Tracking Technical Working Group and the Ministry of Health and Social Services (MOHSS) to strengthen resource tracking - Provided capacity building and mentoring support in hospitals to ensure staff understand RT methodology, approach, and results *Produce and use evidence for advocacy to increase DRM for HIV/AIDS* - Produced efficiency study of hospitals to expand Namibia’s fiscal space for HIV funding	*Build capacity to improve budgeting and execution* - SFI engaged many stakeholders, including policymakers, funding partners, and researchers, to support sustainability planning for HIV - Supported the development of a standardized HIV service package. The Package of HIV/AIDS Services for Epidemic Control provides a concrete way for Namibia’s government to integrate HIV services into its universal health coverage plan. It further supports sustainability by transitioning HIV resources from donor to government financing *Produce and use evidence for advocacy to increase DRM for HIV/AIDS* - Study found that 52 percent of hospitals had technical inefficiencies, presenting opportunities for savings. Addressing the inefficiencies found by this study would save 32 percent in clinical staff costs and 46 percent in recurring purchases	n/a
Cambodia	Health Policy Plus	*Build capacity to improve budgeting and execution* N/A *Produce and use evidence for advocacy to increase DRM for HIV/AIDS* - Worked closely with GFATM, NCHADS and NAA demonstrating need to increase domestic financing for HIV/AIDS, PLHIV considered as vulnerable population, and emphasized importance of CSOs to deliver HIV services; identify and work with policy champions; and planned activities for several years for expected policy changes - Advocacy materials developed with GFATM to showcase the need to increase commitment for procuring ARVs using domestic public resources	*Produce and use evidence for advocacy to increase DRM for HIV/AIDS* - From 2018–2020 the Government of Cambodia invested US$1.5 million per year to procure ARVs until 2019 then incremental increase to US$5 mill by 2023 and maintain investment in subsequent years - Developed Policy Circular, SorChorNor #213, which had six main components that helped to increase the budget allocation for HIV/AIDS, expand PLHIV access to social protection schemes and maintain an effective and sustainable HIV response. Endorsed by the Prime Minister	Potential 35:1
Ethiopia	Health Policy Plus	*Build capacity to improve budgeting and execution* N/A *Produce and use evidence for advocacy to increase DRM for HIV/AIDS* - Baseline assessment of DRM for HIV/AIDS program, and supported with HAPCO, bringing experts together to review - Findings from baseline assessment used to inform the development of the Domestic Resource Mobilization and Sustainability Strategy	*Produce and use evidence for advocacy to increase DRM for HIV/AIDS* - Baseline data used in the development of the DRMS strategy developed - Strategy aims to domestically finance 30 percent of the 2021–2025 National HIV AIDS Strategy Plan. If endorsed, the plan would increase annual federal budgets for HIV from approximately US$700,000 to US$9.1 million and increase annual regional budgets from US$1.2 million to US$7.3 million by 2025 - SFI estimated that Ethiopia could raise US$14 million for HIV programming during 2021–2025 through the integration of a comprehensive package for HIV services into 1,300 Community Care Coalitions (CCCs) in 200 woredas. Ethiopia’s Caring for Vulnerable Children CCCs generate an estimated US$1.2 million annually; scaling up to all kebeles nationally could generate US$39 million annually - Voluntary participation in the AIDS Fund by government and private sector employees could also mobilize considerable resources - SFI also reviewed the existing tax structure in Ethiopia to identify possibilities to improve HIV funding	If DRMS Strategy passed and implemented: potential of more than 46:1 return on investment
Dominican Republic		*Build capacity to improve budgeting and execution* N/A *Produce and use evidence for advocacy to increase DRM for HIV/AIDS* - Generated evidence and provided advocacy efforts to ensure continued funding for the HIV response - Supported non-governmental organizations’ participation in the national health insurance scheme to make the HIV response more financially sustainable - Strengthened the capacities on health workers to increase health insurance enrollment for PLHIV	*Produce and use evidence for advocacy to increase DRM for HIV/AIDS* - This work contributed to the increase in government contributions for ARVs by US$8.4 million in 2019 - SFI promoted inclusion of ARVs in family health insurance benefit packages and recommended a more efficient warehousing of ARVs that will save government an estimated US$163,000 annually - SFI worked to strengthen NGOs’ financial prospects to support the sustainability of the HIV response. Formalizing NGO engagement with the National Health Insurance (Seguro Nacional de Salud, or SENASA, in Spanish) means that participating NGOs receive payments for services rendered to registered PLHIV - SFI worked with government and NGOs to formalize NGO responsibilities for PLHIV enrollment. It did this by building the capacities of NGO staff, providing technical assistance, and facilitating peer learning to improve NGO performance and ensure they followed SENASA procedures. The ripple effect of SFI’s work was significant. SFI trained 140 staff at HIV clinics across the country to enroll people in the national health insurance program, thereby increasing access for PLHIV to the services they need. In only four months between April and August 2018, SENASA enrolled 7,644 PLHIV, a 38 percent increase, in the subsidized regimen. SFI also supported the development of informational brochures on the benefits of enrolling in the subsidized plan that were distributed around the country	n/a
Tanzania		*Build capacity to improve budgeting and execution* - SFI focused on building the capacity of government institutions and civil society organizations to improve how HIV funds are allocated and spent, and how to frame advocacy messages effectively, ensuring continued domestic funding for HIV post-SFI *Produce and use evidence for advocacy to increase DRM for HIV/AIDS* - Budget analysis brief was developed to highlight budget release improvements needed and SFI worked closely with the Government of Tanzania (GOT) for policy change - Cost and efficiency analysis was produced for differentiated service delivery for HIV treatment (facility vs community levels) and potential efficiency gains identified for multi-month dispensing (MMD) and differentiated laboratory management at facility level	*Build capacity to improve budgeting and execution* - Government of Tanzania commitment of US$4.6 million and US$4.5 million for ARVs in 2016/17 and 2017/18, respectively - The National AIDS Control Program to incrementally increase funding for HIV commodities and test kits by 2020 *Produce and use evidence for advocacy to increase DRM for HIV/AIDS* - GOT included in its policy priorities a directive to improve budget release of health and HIV allocations - Contribution led to a new budget allocation of US$114 million per year in 2016 and 2017 to purchase and deliver essential health commodities, which were not in previous budgets. This included a first-ever allocation of US$4.6 million to purchase antiretroviral drugs during the same period - Disbursements of these new budget line items were limited/lower than expected, indicating there is opportunity for improvement through greater investments in budgeting, allocative efficiencies and tracking of expenditures through strengthened public financial management - Total economic cost of ART services could be reduced by US$258 million over a five-year period by using MMD and differentiated facilities - SFI jumpstarted the Government of Tanzania’s efforts to repay its outstanding balance at the Medical Stores Department (including HIV commodity and supply chain costs) with US$5.1 million has been repaid thus far	29:1

### Produce and use evidence for advocacy to increase DRM for HIV/AIDS

PFM systems along with other resources can be used to develop evidence to advocate for increasing government domestic resources for health and HIV/AIDS. In Kenya and Cambodia, evidence was used to advocate to increase or reinstate funding specifically for ARVs, HIV test kits, and other HIV commodities. For both countries, it was important to work closely with The Global Fund so that part of the co-financing goal was focused on procuring HIV commodities as well as to work across government sectors. In the case of Cambodia, SFI supported Cambodia’s National AIDS Authority (NAA) to produce a series of briefs and reports on critical analyses conducted such as estimated costs and benefits to PLHIV from enrolment in social health insurance, assessing the role of CSOs in the HIV response and costs to the government for their services, and highlight ARV co-financing in Cambodia was the lowest in the region [11–13]. Such evidence was used during high-level meetings and to promote the importance of country owned, multisectoral led response to sustain HIV epidemic control during NAA’s Policy Advisory Board – a multisectoral forum.^1^ This led to the development of an advocacy action plan for HIV sustainability that was embedded across the government.

Additionally for Kenya, the timing of this advocacy was critical. During devolution, it was stated by the Government of Kenya that ARVs and other HIV commodities were to be the responsibility of the counties but through advocacy work and the collaboration with The Global Fund, HIV commodities became a national level function again and the policy to procure central medicines was updated. In other countries, such as Ethiopia and Nigeria, evidence was used to develop HIV domestic resource mobilization strategies. The Dominican Republic used evidence to showcase the need to continue funding the HIV response by the government. Vietnam used evidence to show government policymakers that the financial liability for SHI to cover ART is minimal by using SHI funds more strategically. Additionally, evidence was also generated at the provincial level to advocate for mobilizing and allocating local government funding to subsidize SHI premiums and ART copayments for those classified as poor.

### Measuring Impact

SFI emphasized the importance of having measurable impact. Overall, when possible, a return on investment (ROI) was estimated consisting of the increase in health and HIV/AIDS funding and the potential link to the activities on strengthening PFM and evidence-based advocacy divided by the SFI funds used to accomplish those activities. Additionally, specific outcomes of percent change in government budget allocation to health and HIV/AIDS and percent change in government execution of funds for health and HIV/AIDS were also captured annually. Where applicable, the percent change of specific HIV commodity line items was also estimated annually. Other factors may have also influenced the increase in health and HIV/AIDS funds, with possible inflation of the ROI estimates given that there was no counterfactual, such as parallel activities to strengthen the systems-side of PFM by country governments and other organizations, absorptive capacity, co-financing requirements set by multilateral institutions (such as the one set by the Global Fund to Fight HIV/AIDS, Tuberculosis, and Malaria), etc. Despite such limitations, SFI was still a pioneer in attempting to create, albeit imperfect, measurement of financial impact given estimated inputs. Limitations of the estimated ROIs are discussed in more detail under Lessons Learned.

## Results

Results for the different country program implementation are specified in Table 1. Over the 8 years, SFI implementation for improving health and HIV/AIDS domestic government mobilization, budget allocation, and execution was met with much success but also a few challenges.

### Build capacity to increase domestic government resource mobilization, improve budget absorption, and maximize resource use

Program implementation in five out of the nine countries that focused on building capacity to improve budgeting and execution of health and HIV/AIDS funds led to clear increases in budget allocation and spending. In Kenya, the average county government allocation to health increased from 23% in 2015/16 to 27% in 2017/18, compared to 13% prior to devolution (2013) [14, 15]. Budget execution rates improved from 73% in 2016/17 to 84% in 2018/19 for deep dive counties [15]. At the county level, an increase of US$180 million and US$8.7 million was mobilized and spent on health and HIV, respectively, from 2015/16 to 2018/19 [16]. Domestic funds for health and HIV continue to increase annually, with continued improvements in budget absorption demonstrating the lasting effects of SFI’s effort. Uganda increased budget execution rates at the Ministry of Health (MoH) from 80% in 2015/16 to 97% in 2017/18, leading to an additional US$17.4 million in health spending [17]. Uganda’s Revenue Authority increased debt collection in tax arrears by 50% from UGX 49 billion in 2017 to UGX 73 billion Uganda in 2018, an increase of almost US$7 million in revenue [17]. In Vietnam, budget execution and SHI commitments for ARVs increased by US$15.4 million, while local governments committed US$2.4 million to subsidize SHI premiums and copayments for PLHIV [18]. This contributed to an increase in domestic government resources for HIV from less than 30% in 2015 up to more than 50% by the end of 2020. Further commitments were made by half of the provinces to mobilize funds for SHI premiums and ARV copayments. In Nigeria, Lagos and Kano experienced a US$1.7 million and US$1 million increase in health spending, respectively [19]. Additionally, Lagos allocated US$1.7 million for HIV/AIDS between 2019 and 2020. The work in Rivers was halted due to slow progress and funding limitations despite project efforts and therefore efforts were shifted to just focus on Kano and Lagos. In Tanzania, the National AIDS Control Program was to contribute, for the first time, resources for ARVs. Commitment from the Tanzanian Government was for US$4.6 in 2016/17 and US$4.5 million in 2017/18, with plans for incremental increases in funding for ARVs by 2020 [20]. Ultimately the proposed funds for ARVs were spent on strengthening the country’s supply chain instead with a focus on storage and distribution. ARVs remain to be fully funded by donors in Tanzania.

The remaining four countries that focused on capacity building were on activities to improve resource allocation, but concrete outcomes of such work could not be captured. Sometimes this was due to the type of work that was conducted (e.g., support future HIV sustainability planning) and other times it was due to results being lagged by a year given reporting cycles on spending resources at the end of a fiscal year. In Botswana, three training-of-trainers (ToT) health resource tracking workshops were held; training a total of 46 key national and regional staff, with the goal of cascading the training down but led by the ToTs after the project was completed [21]. Unfortunately, the procurement demands for addressing COVID-19 led to a fewer number of finance, accounts, and procurement officers trained. The work in Nepal continued to be delayed because of lockdowns early in the pandemic. This led to the budget advocacy training for national and provisional CSOs occurring after the 2020/21 budget cycle. Nonetheless, the positive feedback from the CSOs of the training, demonstrated improvement in knowledge of budgeting among the 28 national and 24 local CSOs, and commitments from CSOs and government officials to prioritize HIV interventions and increase funding for the upcoming fiscal year highlights the importance of such trainings and usefulness of simple tools to support CSOs advocacy work for years to come [22].

The work in Namibia paved the way for the government to integrate HIV services into its universal health coverage program, which emphasizes an increased role of government financing [23]. Lastly, in September 2020 Côte d’Ivoire organized two training good governance workshops with participants including representatives from the General Directorate of Health, the National HIV Control Program, and representatives of the regional and departmental health system. Participants were presented with a tool to monitor budgeting and expenditure (known as BOOST). The Implementing mechanism was unable to continue the activities initiated with the Ministry of Health and Public Hygiene due to reduced budgets. However, the 2021–2025 national health development plan (PNDS 2021–2025) currently being validated includes the BOOST tool at the central level of the Ministry of Health, Public Hygiene and Universal Health Coverage. Although specific increases in HIV budgets and improvements in execution or efficiency gains could not be demonstrated at the end of these programs, like in Nepal, it will be continuously assessed through the Resource Alignment initiative led by PEPFAR that aligns PEPFAR, Global Fund, and host country governments budget and expenditure line items. In other countries, like Namibia, it was more challenging to show attribution because the work accomplished was higher-level policy work but should support future sustainability of their HIV/AIDS program.

### Produce and use evidence for advocacy

**Fig. 1:**
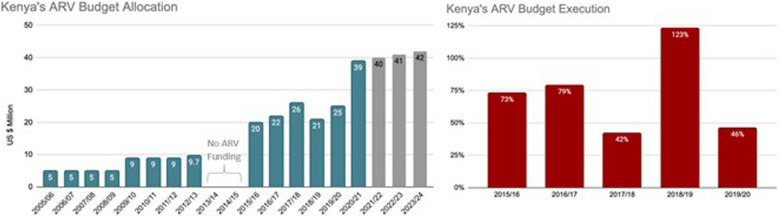
National Kenya Government ARV Budget Allocation Over Time

Eight SFI countries focused on using evidence to advocate for more domestic resources for health and HIV/AIDS from government budgets, ensure the execution of those funds, and highlight areas for efficiency gains. Post-devolution in Kenya, there were no government funds for ARVs in 2013/14 and 2014/15. Through the advocacy work mentioned above, a budget line was reinstated, restoring government funding for ARVs that has only grown over the years from US$20 million to US$39 million; and given the government commitments will continue to grow over the next several years (see Fig. 1) [24, 25]. Despite this success, the execution of these additional funds was met with continued procurement challenges and thus erratic and sometimes extremely low budget absorption of the government ARV funds (Fig. 1). In the DR, government contribution for ARVs increased by US$8.4 million in 2019 [26].

**Fig. 2:**
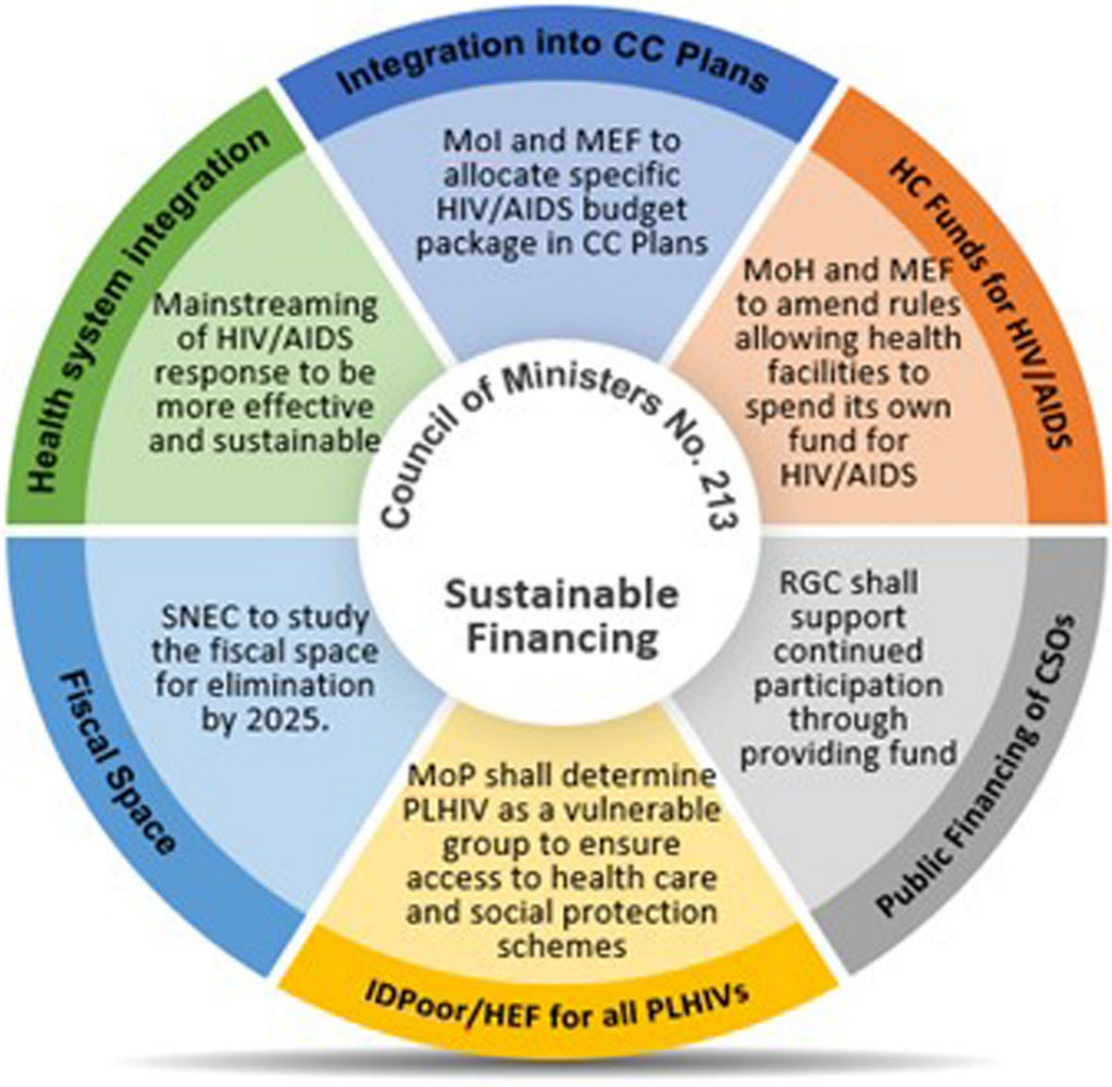
Cambodia’s Sustainable HIV/AIDS Financing Policy Circular—SorChorNor#213

Policy advancement is possible in a more challenging political environment as demonstrated in Cambodia. Identified policy champions, backed by sustained technical support from SFI over the medium-term, were found to be a critical component of bringing about real change to the HIV funding landscape in-country. Cambodia’s government elevated the importance of its HIV/AIDS program, leading to an increase in financial commitments for ARVs from US$1.5 million annually from 2018–2020 to incremental increases to US$5 million annually by 2023 and to maintain such an investment in subsequent years [27]. In addition to the government’s financial commitments to procure ARVs, the Prime Minister endorsed a policy circular (SorChorNor#213), highlighting the government’s approach to combat the HIV/AIDS epidemic (see Fig. 2). This circular has six components (see Fig. 2) that increase the government budget for HIV/AIDS, expand people living with HIV (PLHIV) access to social protection schemes (like the Health Equity Fund—making all healthcare free for PLHIV), and maintain an effective and sustainable HIV response [28].

Both Nigeria and Ethiopia focused on developing a domestic resource mobilization strategy for HIV/AIDS. If passed in Ethiopia, the strategy envisages to domestically finance 30% of the 2021–2025 National HIV/AIDS Strategy Plan and would increase the national budget for HIV/AIDS from US$700,000 to US$9.1 million and regional budgets from US$1.2 million to US$7.3 million by 2025 [29]. The implementation of this strategy will be made once endorsed by the Council of Ministers and implemented. In Vietnam, the success of advocacy efforts in getting the government commitment to make SHI the main source of funding for HIV treatment and to subsidize SHI premiums and ARV copays for those in need. Its efforts have resulted in more DRM for the HIV program from 36% of total in 2017 to 51% in 2020 and it will be increasing from 2023 onward [18]. The review of the current HIV strategy 2016–2020 and development of the new 2021–2030 HIV strategy used costing evidence and financial analysis from SFI to estimate resource needs, potential resource gap, and recommendations for priority areas of the HIV program (e.g., outreach, community testing and PrEP). The Nigerian government finalized and endorsed the DRMS in June 2021. This strategy will mobilize up to US$662 million for HIV response domestically within the next 4 years [30].

SFI’s efforts in Tanzania led to a new budget allocation of US$114 million per year in 2016 and 2017 to purchase and deliver essential health commodities, which included a first-ever allocation of US$4.5 million to purchase ARVs during the same period [20]. Like many other countries, an increase in budget allocation does not directly reflect an increase in spending where disbursement of the new budget line items was limited. However, a budget analysis brief produced by SFI did lead to the Government of Tanzania to include in its policy priorities a directive to improve budget releases of health and HIV/AIDS allocations. Efficiency gains in service delivery, systems, and processes also frees up resources to be reinvested into health and HIV/AIDS programs. The promotion of including ARVs in the family health insurance benefits package in the DR led to an estimated savings of US$163,000 annually through more efficient procurement processes [26]. In Tanzania, a study produced by SFI found that multi-month dispensing (MMD) and differentiated care models could reduce the total service delivery cost of ART services by US$258 million over a five-year period [20]. Lastly, in Namibia an efficiency study found that 52 percent of hospitals had technical inefficiencies, and if addressed that these facilities could save 32 percent in clinical staff costs and 46 percent in recurring purchases [23]. However, follow-up assessment is needed to determine quality of health care services within these facilities because technical efficiency analysis only compares the relative efficiency in resource use from one hospital to another regardless of stock-outs experienced, protocols followed, etc.—factors that contribute to quality health care.

### Measuring Impact

Many of the SFI-supported countries saw a positive return on investment (ROI), although other factors may have also attributed to this, when assessing the cost of the project relative to the amount of funds increased for health and HIV/AIDS in a country. Kenya had the largest estimated ROI, of almost sixty dollars mobilized and expended for every dollar invested by SFI (59.7:1). This was followed by Nigeria (36:1), Tanzania (29:1), Vietnam (19:1), and Uganda (9:1). Ethiopia and Cambodia have a high potential of return on investment if strategies and policies are implemented and commitments realized. An ROI could not be estimated for some SFI-supported activities due to lag time between capacity building efforts and budgeting processes (Nepal) or at least some of the work was high level policy work that is challenging to make a link between such efforts and changes in budget allocations and spending (Namibia and DR).

## Lessons learned

Lessons learned from this work are multifold: 1) Robust data is needed to provide the evidence to inform how to strengthen PFM systems; improve transparency of spending for host country governments, and ultimately increase government domestic resources for health and the HIV response; 2) Institutionalizing capacity building efforts will allow continued technical assistance (TA) long after program implementation; 3) Policy-related work should be considered over at least the medium-term, where it takes several years for government commitment and policy changes to occur but will be rewarding in the long-run; 4) PFM success can be stymied by political transitions, political will, and donor commitments; 5) COVID-19 brought new challenges but also new opportunities into potentially more efficient programming; 6) Emphasizing measurable results targets investments for greater impact; and 7) Results are not necessarily solely project attributions, where other factors may influence domestic resource mobilization for health and HIV/AIDS and spending the funds.

### Robust data needed to strengthen PFM systems and increase domestic government health and HIV resources

The last few years, efforts to harmonize budgets and expenditures for PEPFAR, Global Fund to Fight AIDS, Tuberculosis, and Malaria (GFATM), and host country governments to provide greater visibility on total HIV resources for a specific PEPFAR-supported country. This strengthens joint planning, drives efficiencies, and avoids duplication of resources budgeted and spent between two major donors. It additionally provides much needed evidence to link HIV resources with program indicators.

More recently, global health institutions—Office of the U.S. Global AIDS Coordinator and Health Diplomacy (OGAC), USAID, GFATM, UNAIDS, and others—rallied around implementing a unified approach to capture the real costs of a country’s HIV response more routinely. This initiative called Activity Based Costing and Management (ABC/M), supports the need to align and optimize investments for a sustainable transition of a country’s HIV program with a focus on making data collection and use for more effective decision-making more routine and integrated into existing government systems [31, 32]. It provides a holistic approach by including facility, community, above-site levels to capture the real costs of a country’s HIV program more routinely. To date, USAID provides technical leadership in the overall approach and implementation in Kenya, Mozambique, and Namibia. SFI spearheaded the implementation of ABC/M by financially supporting and managing the implementation of the first two countries – Tanzania and Uganda.

The two initiatives mentioned above along with other efforts such as the responsibility matrix, sustainability dashboard index by PEPFAR will be used to determine funding along various aspects of the HIV program and support advocating and monitoring the increase in domestic government resources.

Data needs to inform how best to strengthen PFM such as identifying bottlenecks and root causes to determine possible solutions; improving transparency and accountability of government allocations and spending; and showcasing funding needs for health and HIV/AIDS. Strengthening in-country PFM systems will provide governments with the data and tools to enhance budgeting and planning for a more sustainable HIV program.

### Institutionalizing PFM capacity building ensures continued TA post-program implementation

One-off training or technical assistance support will not entirely address the problems of limited capacity in the PFM systems established in-country, especially when countries are faced with high government staff turnover. Ensuring that capacity building efforts continue when a project ends, it is imperative that such efforts are institutionalized through curriculum development, a training of trainers program from reputable local institutions/universities and mentoring or coaching program for the trainers while training is rolled out. Empowering local institutions to lead the capacity building efforts promotes a country’s ability to provide the necessary technical support to continue enhancing PFM systems and the use of data to ensure adequate funding for health and HIV/AIDS through increased DRM and/or efficiency gains furthering a sustainable financing agenda for HIV/AIDS.

### Policy changes: A medium-term goal

Policy work, along with system strengthening, does not occur overnight. As highlighted in Uganda, a “Big Bang” approach to Program-based budgeting (PBB) implementation is not a practical approach. Instilling policy change takes years of effort, not just a few months or even a year. When developing work plan timelines, projects need to be mindful of the time it takes to cultivate relationships and trust with political and technical leadership, work with them to highlight areas for change, and then go through the government system(s) for change. Although policy reform takes time and effort, if done in the right way, the outcome can be effective and sustainable. This becomes especially challenging when working with donor funding that is on an annual budget cycle such as PEPFAR with the COP/ROP process.

SFI has demonstrated that if a clear plan is developed and indicators of measurement are developed, it is possible to reap the rewards of funding medium-term policy work. A clear medium-term (3–5 years) strategic plan with measurable results for each year is needed to produce desired policy changes. Even with a plan of action, little movement will occur unless there is an identified champion who believes and supports the objectives that a team could coach and provide evidence and other materials to make a case for a policy change. Cambodia’s success in the Prime Minister endorsing the policy circular was because the SFI team identified a champion that strongly supported a more holistic approach to combating HIV/AIDS. Vietnam is another example when SFI worked alongside a committed champion at the MOH to advocate for a special Prime Minister Decision that paved the way for provincial budget allocation, execution, and mobilization of provincial funds for the poor to subsidize premiums and ART copays for HIV patients. For any policy work, identifying and using champions can bring projects to the forefront of the political agenda. It is important to accurately identify the right champion(s) for the task who are willing and able to support advocacy efforts, are technically supported, and their time managed appropriately.

### PFM success can be stymied

It takes time to achieve success in PFM reforms, and therefore is susceptible to shocks such as changes in political leadership. Political transitions are inevitable and yet can still threaten the success of a PFM system strengthening if a new party in power de-emphasizes the need for transparency, accountability, and evidence to make decisions. Those working in this space may have to start over to build relationships and trust with a new government to ensure that the political will remains for stronger PFM systems. On the other hand, with the right mix of timing and political will, strengthening PFM systems can bring a lot of reward for investments made. This was the case in Kenya where the SFI support on PFM was at a critical point post-devolution which led to a rapid increase in health and HIV/AIDS domestic spending to improve outcomes. Furthermore, going beyond commitments to increase government contributions to health, HIV, or even specific commodities will lead to the allocation and execution of financial commitments made. Creating a specific line item for commodities or types of services will limit potential reallocations of funds during the budgeting process. Reinstituting a line item for ARVs in the national budget of Kenya led to a steady increase in resources allocated to ARVs over the last six years; whereas in Tanzania, financial commitments for procuring ARVs fell short as funds were redirected to supply chain support. As countries prepare for transitioning from donor support, instituting line items for specific HIV services and commodities in the national and sub-national budgets could ensure the sustainability of the HIV program.

Increased allocations do not always translate into increased spending, and this can have spillover effects in raising funds through different avenues other than tax revenue. In Tanzania, delays in national government budget release hindered health service delivery and this reduced the private sector’s confidence to contribute funds to a Government of Tanzania institution such as the AIDS Trust Fund [20].

It is important to work closely with other major donors when bringing forward evidence to a country’s government for greater DRM. If overlooked, then it is possible that other donors may fill the financial gap. Although this would be welcomed by country governments with finite resources, it becomes a temporary fix to focusing on long-term sustainability.

### COVID-19: New challenges and new opportunities

Prior to the COVID-19 pandemic many countries were on track to reaching epidemic control and governments made strong financial commitments towards the fight against HIV/AIDS. The work done under SFI contributed to ensuring that these financial commitments came into fruition and that the resources allocated were spent timely. Adaptability and resiliency of projects is key, which has become more evident with the new normal that we are currently operating under because of the COVID-19 pandemic. Although this is sometimes potentially challenging with donor funding, allowing projects the flexibility to pivot when unforeseen circumstances arise will allow for a more rewarding outcome(s).

The COVID-19 pandemic has changed the projected economic growth for most countries, and globally, and escalated health spending specifically for COVID-19 prevention, care, and treatment [9]. Innovations and the increasing need to leverage the private sector has staved off some of the potential negative externalities that COVID-19 posed for PLHIV in continuing their needed care and treatment. This is explored further in the SFI PSE paper of the SFI supplement [33]. With the emphasis on health care specifically to tackle COVID-19, government resource allocation for HIV/AIDS has been lower than previous commitments. Nonetheless, reducing bottlenecks have led to improved execution rates of these funds, and ultimately health outcomes and the focus on efficiency has become even more important [10, 34]. This is even more important with the slowed economic growth experienced globally because of the COVID-19 pandemic despite the expected rebound for many countries this year and the next if new variants do not cause new waves of outbreaks and additional constricting economic measures [9]. Despite all the unforeseen challenges that the pandemic presented, SFI was still able to deliver results and continue to work with governments to ensure that financial commitments to HIV services remained.

### Emphasizing measurable results targets investments for greater impact

Oftentimes, policy work especially related to health financing has been challenging to measure results. This is partially due to other factors that influence health financing efforts that might be beyond a project’s control. SFI set out to identify measurable results with clear indicators of measurement. This allowed teams to measure progress and course correct if a particular activity was not producing the desired results to better target investments when needed. This is what occurred in Rivers, Nigeria where challenges hindered progress. With the team unable to reach certain measurable indicators, it was agreed to refocus efforts in the two states that were showing progress was being made. The emphasis on measurable results has allowed SFI to bring greater impact in the investments made on PFM, as evident in the estimated ROIs, and has held teams to a higher standard of project outcomes.

### Results contributions, not attributions

Caution is needed when reporting results from PFM strengthening or resource mobilization advocacy programs of work. Other factors like political will or other agencies supporting system strengthening also influence domestic resource mobilization for health and HIV/AIDS, the absorption of spending these funds, and efficiency gains made. It is important to understand that correlation does not necessarily mean causation. For some of the activities presented in this paper no ROI could be estimated, whereas for other activities the ROIs presented might be higher due to these other factors contributing to increased spending and efficiency improvements for a country’s HIV/AIDS program. Also, teasing out the ROI by pillar—especially between PFM and financial protection [35]—was challenging at times when increases in government contributions for ARVs and other commodities was done through the social health insurance schemes or to implement decentralized drug distribution [33]. Nonetheless, SFI demonstrates that it is possible to measure the link between effort and financial impact despite some of these caveats in the degree of the ROI estimated.

## Conclusion

Strengthening PFM systems, evidence-based advocacy for increased domestic resource mobilization, and use of evidence for policy changes and decisions has shown positive results to increase government financing for health and HIV. Although, as in the case with any PFM activity, it is challenging to demonstrate causation between activities and outcomes. SFI is the first known government program that measures impact in financial terms with estimated return on investments. Although imperfect, it paves the way for others to reconsider how to measure impact more effectively in how donor resources could leverage greater impact. This paper highlights an effective approach to unlock sustainable resources that can be spent more effectively to address a country’s health and HIV response, closing the investment gap needed to end the HIV/AIDS epidemic by 2030.

## Data Availability

The datasets used and/or analyzed are available from the corresponding author on reasonable request.

## Abbreviations

PFM: Public financial management
DRM: Domestic resource mobilization
DRMS: Domestic resource mobilization and sustainability
HIV: Human immunodeficiency virus
AIDS: Acquired immunodeficiency syndrome
SFI: Sustainable financing initiative
PEPFAR: President’s emergency plan for AIDS relief
ROI: Return on investment
COP/ROP: Country operational plan/regional operational plan
MOH: Ministry of health
GFATM: Global fund to flight AIDS, tuberculosis, and malaria
TA: Technical assistance
ARV: Antiretroviral
ART: Antiretroviral therapy
PLHIV: People living with HIV
ToT: Training of trainers
CSO: Civil society organization
SHI: Social health insurance
IMF: International monetary fund
USAID: United States Agency for International Development

## Footnotes

1 The NAA Policy Advisory Board consists of representatives from the Ministry of Health, Ministry of Economy and Finance, Ministry of Defense, Ministry of Planning, and Ministry of Interior and is chaired by NAA. The goal of this board is to discuss critical HIV priorities, present new evidence, and achieve consensus on key policy measures and resolutions related specific roles of each Ministry in Cambodia’s efforts to sustain the HIV response.
